# Determination of microbiota awareness levels in women planning pregnancy

**DOI:** 10.1590/1806-9282.20231401

**Published:** 2024-05-20

**Authors:** Rabia Atay, Ozgenur Hacioglu

**Affiliations:** 1 Kirklareli University, Institute of Health Sciences, Department of Midwifery – Kırklareli, Turkey.; 2 Kirklareli University, Faculty of Health Sciences, Department of Midwifery – Kırklareli, Turkey.

**Keywords:** Microbiota, Awareness, Women, Pregnancy

## Abstract

**OBJECTIVE:**

It was recently discovered that the microbiota has a significant impact on pregnancy, gynecological, and neonatal health. However, studies indicate that people struggle to understand topics, such as microbiota, microbiome, probiotics, and prebiotics, or comprehend them inaccurately or incompletely. Understanding the human microbiota and probiotics that can regulate the microbiota helps women develop daily habits for both healthy nutrition and health protection. The aim of this study was to assess the microbiota awareness levels of women who are planning pregnancy.

**METHODS:**

A cross-sectional descriptive study was carried out on 417 women who were planning pregnancy. Face-to-face interviews and questionnaires were used to collect research data. A microbiota awareness scale was used as a data collection tool.

**RESULTS:**

The study found a statistically significant difference in the subdimension scores related to microbiota awareness, general information, product knowledge, chronic disease, and probiotic and prebiotic knowledge based on the educational status of the participants. The study concluded that the participants had a confusion about microbiota awareness, general information, product information, chronic disease, and probiotic and prebiotic subdimensions. Furthermore, it was found that the participants had only a partial understanding of the relationship between microbiota and diseases.

**CONCLUSION:**

It is recommended that training programs focusing on the relationship between microbiota and health in women, such as "microbiota and its importance in women's health" and "microbiota and disease relationship," be organized and women would be encouraged to participate in these training programs.

## INTRODUCTION

The term "microbiota" is derived from ancient Greek words "micro" (μικρος, small) and "biota" (βιοτα), which refers to the living organisms of an ecosystem or a specific area^1^. Microbiota refers to all microorganisms present in different parts of our body, while microbiome refers to their genetic materials^2,3^. Our "last organ" is known as the human microbiome^4^. In recent years, researchers have begun to understand the effects of microbiota on pregnancy, the postnatal period, infant health, obesity, allergic diseases, gastrointestinal system, and urogenital infections such as vulvovaginal infection (VVI) and bacterial vaginosis (BV) infections^2-5^.

The mode of delivery plays a significant role in determining the microbiota makeup of newborns. While babies delivered vaginally are exposed to the mother's vaginal and intestinal microorganisms, babies delivered through cesarean section are exposed to the mother's skin and environmental microorganisms. Various factors such as prenatal probiotic use, gestational week at birth, frequency of vaginal examination, birth environment, birth weight, length of hospital stay after birth, baby's diet, economic level, number of siblings, geographical location, climate, culture, gender, country's development level, and perinatal stress factors all influence newborn gut microbial diversity and colonization^6^.

Pregnancy is a crucial period in a woman's life that involves various immunological and metabolic changes. The most important factors that influence the formation of a healthy microbiota in a child are the transfer of microbiota from mother to newborn during birth and the continuation of these processes with breastfeeding^7^. Given the importance of microbiota in pregnancy and women's, and children's health, knowing the microbiota of women planning pregnancy and concepts such as probiotics that can modulate their microbiota helps them develop daily habits for both healthy nutrition and health protection. However, there is a lack of studies aimed at determining the level of awareness about microbiota in individuals. Therefore, this study aims to determine the level of awareness about microbiota among women planning pregnancy, which can help in education planning based on their needs for this purpose.

## METHODS

This study was conducted at Kirklareli Training and Research Hospital between February 2022 and July 2022. The population for this study included 417 female participants aged 18 and higher with no communication problems and who applied to the hospital during the research period. The participants agreed to take part in the study and signed the informed consent form.

### Data collection tools

The data collection tool is separated into two sections. The first section includes questions related to the individuals' sociodemographic traits, while the second section consists of the microbiome awareness scale form comprising 28 items^8^.

### Evaluation of data

The SPSS (Statistical Package for Social Sciences) 25.0 program was used to analyze the study data. The independent sample t-test and one-way analysis of variance were also used. Statistical significance was accepted as p<0.05.

### Ethical aspect

The study was conducted with permissions of the Kirklareli University Health Sciences Institute Ethics Committee (Number: PR0366R01) and the Kirklareli Provincial Health Directorate (Number: 2, February 8, 2022). After the purpose of the study was explained to the participants, written and verbal agreements were obtained. Additionally, permission to use the scale was obtained from the authors who created the scale used in the study. All practices were carried out in accordance with the 1964 Helsinki Declaration.

## RESULTS

When the highest distribution rates are evaluated according to the sociodemographic characteristics of the participants, it was determined that 25.4% of the participants' age was 25–30 years and 24.2% of the educational status was associate's degree. It was revealed that 75.8% of the participants stated they had regular and adequate eating habits, while 76% had no chronic diseases. When the smoking status of the participants was investigated, it was found that 56.4% answered that they had never smoked (Table 1).

**Table 1:** Sociodemographic characteristics of the participants.

Variables		n	%
Age (years)	Under 25	51	12.2
	25–30	106	25.4
	31–35	77	18.5
	36–40	89	21.3
	41+	94	22.5
Educational status	Not literate/literate	25	6.0
	Primary school	54	12.9
	Secondary school	70	16.8
	High school	99	23.7
	Associate's degree	101	24.2
	Bachelor's degree	46	11.0
	Postgraduate degree	22	5.3
Location	Provincial center	304	72.9
	District center	91	21.8
	Village	22	5.3
Number of people living in the household (including you)	2	72	17.3
	3	135	32.4
	4	133	31.9
	5	53	12.7
	6+	24	5.8
Number of pregnancy	0	64	15.3
	1	113	27.1
	2	138	33.1
	3	65	15.6
	4+	37	8.9
Number of living children	0	82	19.7
	1	133	31.9
	2	148	35.5
	3+	54	12.9
Do you think that you have regular and adequate eating habits?	Yes	316	75.8
	No	101	24.2
Do you have any chronic disease?	Yes	100	24.0
	No	317	76.0
Total		417	100.0

There was a statistically significant differences in the subdimension scores such as microbiota awareness, product knowledge, and probiotic and prebiotic knowledge based on the participants' age (p<0.05). Participants under the age of 25 years had lower scores in all three subdimensions. Similarly, there was a statistically significant difference in scores observed for subdimensions such as microbiota awareness, general information, product knowledge, chronic disease, and probiotic and prebiotic knowledge based on the participants' educational status (p<0.05). It was observed that participants with postgraduate degrees had higher microbiota awareness scores, while those with chronic diseases had higher scores in product knowledge subdimension than those without chronic diseases (Table 2).

**Table 2:** Comparison of microbiota awareness scale and subdimension scores according to the sociodemographic characteristics of the participants.

Variables		Microbiota awareness (total)	General information	Product knowledge	Chronic disease	Probiotic and prebiotic knowledge
		$\bar{\text{X}}\pm \text{SD}$	$\bar{\text{X}}\pm \text{SD}$	$\bar{\text{X}}\pm \text{SD}$	$\bar{\text{X}}\pm \text{SD}$	$\bar{\text{X}}\pm \text{SD}$
Age (years)	Under 25	63.14±11.18	21.67±4.52	8.88±3.1	15.84±3.32	16.75±3.48
	25–30	66.80±11.87	22.74±4.65	9.68±3.43	16.31±3.12	18.08±4.14
	31–35	70.31±10.16	23.73±4.28	10.69±3.47	17.03±2.66	18.87±3.2
	36–40	69.38±10.36	23.34±3.95	10.61±3.69	16.88±2.63	18.56±3.44
	41+	69.72±11.36	23.33±4.78	11.07±3.36	16.44±2.58	18.88±3.61
	F test	**4.502**	1.987	**4.631**	1.838	**3.614**
	p	**0.001**	0.096	**0.001**	0.121	**0.007**
	Bonferroni	**3,4,5>1**	–	**3,4,5>1**	–	**3,4,5>1**
Educational status	Not literate/literate	62.88±8.71	21.52±3.95	8.56±3.14	16.00±3.01	16.8±2.68
	Primary school	61.26±10.38	21.19±3.98	8.22±2.6	15.52±2.91	16.33±3.76
	Secondary school	65.99±9.65	21.97±4.13	9.9±3.44	16.24±2.49	17.87±2.98
	High school	67.72±10.69	22.66±4.56	10.24±3.16	16.53±2.63	18.29±3.42
	Associate's degree	70.12±11.64	24.01±4.79	10.74±3.51	16.35±2.98	19.02±4.1
	Bachelor's degree	75.30±8.67	25.2±2.68	12.15±3.48	18.00±2.80	19.96±3.29
	Postgraduate degree	77.05±10.57	25.68±4.89	12.64±3.86	18.41±2.54	20.32±3.31
	F test	**12.438**	**7.242**	**9.415**	**5.392**	**7.328**
	p	**0.000**	**0.000**	**0.000**	**0.000**	**0.000**
	Bonferroni	**6,7>1,2,3,4** **5>1,2** **4>2**	**6,7>1,2,3,4** **5>2**	**6,7>1,2,3,4** **4,5>2**	**6,7>2,3,5**	**6>1,2,3** **7>1,2** **4,5>2**

p<0.05; t-test: independent sample t-test; F test: one-way analysis of variance (ANOVA).

X±SD: average (X) and standard deviation (SD).

The answers given by participants to the propositions in the microbiota awareness scale are as stated in Table 3.

**Table 3:** Microbiota awareness scale.

Statements*	I strongly disagree		I do not agree		I'm undecided		I agree		Absolutely I agree	
	n	%	n	%	n	%	n	%	n	%
1	21	5.0	16	3.8	31	7.4	226	54.2	123	29.5
2	13	3.1	22	5.3	113	27.1	201	48.2	68	16.3
3	19	4.6	38	9.1	93	22.3	180	43.2	87	20.9
4	20	4.8	26	6.2	86	20.6	180	43.2	105	25.2
5	11	2.6	32	7.7	129	30.9	180	43.2	65	15.6
6	24	5.8	18	4.3	41	9.8	202	48.4	132	31.7
7	22	5.3	28	6.7	98	23.5	193	46.3	76	18.2
8	13	3.1	33	7.9	209	50.1	138	33.1	24	5.8
9	20	4.8	27	6.5	81	19.4	206	49.4	83	19.9
10	18	4.3	36	8.6	173	41.5	157	37.6	33	7.9
11	13	3.1	32	7.7	132	31.7	185	44.4	55	13.2
12	17	4.1	26	6.2	198	47.5	146	35.0	30	7.2
13	23	5.5	19	4.6	47	11.3	168	40.3	160	38.4
14	8	1.9	25	6.0	239	57.3	111	26.6	34	8.2
15	18	4.3	19	4.6	106	25.4	191	45.8	83	19.9
16	26	6.2	44	10.6	205	49.2	110	26.4	32	7.7

* Statements: (1) The human body contains a large number of microorganisms. (2) The gut microbiota begins to form when the baby is in the womb. (3) I know what prebiotic products are. (4) The use of antibiotics adversely affects the intestinal microbiota. (5) Disruptions in the intestinal microbiota cause obesity. (6) Diet is one of the important factors affecting the intestinal microbiota. (7) I know what probiotic products are. (8) Changes in the microbiota are associated with bowel cancer. (9) Probiotics should be consumed regularly. (10) Disruptions in the intestinal microbiota cause diabetes. (11) I believe that using probiotics can help with diarrhea. (12) An increase in the number of harmful bacteria in the intestines can cause nonalcoholic fatty liver disease. (13) Breastfeeding positively affects the intestinal microbiota of the baby. (14) Changes in the gut microbiota are associated with celiac disease. (15) I believe that using probiotics can help with constipation. (16) There is a link between gut microbiota and depression and Alzheimer's.

## DISCUSSION

The vaginal microbiota, which is part of the female microbiome, has crucial functions. It is made up of a balanced host of *Lactobacillus* bacteria, anaerobic bacteria, and *Candida* yeast. However, in some situations, these microorganisms exhibit dysbiosis, which can lead to vaginal infections^2^. *Lactobacillus* bacteria, which dominate the vaginal microbiota of healthy women, maintain vaginal pH and inhibit pathogen growth by secreting numerous antimicrobial compounds. Therefore, the dominance of these probiotic bacteria, which protect the vaginal microbiota through various mechanisms, is essential for protecting against recurrent vulvovaginal infection (RVVI), including recurrent vulvovaginal candidiasis (RVVC) and BV infections^9^. Recent studies have clearly demonstrated the importance of microbiota in reproductive system infections, fertilization, pregnancy, postpartum period, and newborn health^5-7^. The newborn's health, which is closely related to women's health, will be preserved, as will the continuity of healthy individuals. When women's health is considered holistically, a woman's overall health will contribute to the pregnancy processes. Because the changes in the pregnancy process make women more sensitive, it is critical to explain the changes that will occur in their bodies and raise their awareness of healthy living. Pregnancy involves numerous processes that contribute to the neonatal microbiota^7^.

Recent studies have highlighted the importance of monitoring individuals' awareness of microbiota and probiotics that alter microbiota^8,10-17^. Researchers noted that people who are aware of the importance of microbiota in their health are more likely to choose probiotic-containing diets^12,13,15^. Similarly, it has been found that people understand that antibiotics lower the amount of beneficial bacteria in the microbiome and that the need to use antibiotics when necessary^18^.

Individuals' awareness of the microbiota–health relationship is also related to their recognition and understanding of microbes. As a consequence, individuals' basic descriptions of microbes should be maintained. Individuals may benefit from this awareness by incorporating components that affect the microbiota into their daily routine, such as nutrition, good hygiene habits, and regular exercise^14^.

When studies on microbiota knowledge are analyzed, it is notable that the majority of the studies assessed the individuals' awareness of the concepts of probiotic, prebiotic, as well as their probiotic and prebiotic food consumption status^10-19^ and directly measured individuals' microbiome awareness^8,13^. It can be noted that the main themes examined are persons of all ages and professional groupings, such as healthcare professionals^14,17^. The same topics were explored utilizing mothers^16^, postmenopausal women^11^, and educational level factors^10-19^.

The microbiome awareness of women planning pregnancy was assessed in this descriptive cross-sectional study. The findings of 417 female participants were included in this study, and the answers given to the questions in the microbiota and awareness scale were discussed by comparing the demographic characteristics of the participants and the scale's subdimensions. Researchers evaluated that the prevalence of probiotic use was higher in female participants with higher education levels^10^. Similarly, in this study, based on the participants' educational status, there was a statistically significant difference in subdimension scores such as microbiota awareness, general information, product knowledge, chronic disease, and probiotic and prebiotic knowledge. The current rates exemplify the awareness among participants that probiotics have important health functions. The effective use of probiotic prebiotics, especially in constipation and diarrhea problems, can be attributed to the fact that they were previously recommended to the participants by physicians. In a study that assessed the probiotic knowledge level and consumption status of postmenopausal women^11^, they enrolled 150 women with ages ranging from 50 to 70 years. Probiotic users (58.9%) were found to be more educated, more aware of gut health, and to have healthier lifestyle habits overall. Another study concluded that participants' education levels and probiotic knowledge levels were related^12^. The level of microbial awareness is affected by educational status. As a result, prospective moms should receive appropriate knowledge and supportive treatment, particularly during the pre-pregnancy phase. Participants in the study were included in the microbiota awareness scale. Finally, the study concluded that the participants had a confusion regarding general information, product information, chronic disease, and probiotic and prebiotic subdimensions, and that they could not establish the relationship between microbiota and diseases such as Alzheimer's disease, celiac disease, obesity, diabetes, and bowel cancer. Similarly, several studies have highlighted that people lack understanding about the relationship between microbiome and diseases^8,11,13,16^.

## CONCLUSION

In this study, it was concluded that the participants experienced confusion regarding general information, product information, chronic disease, and probiotic and prebiotic subdimensions in the microbiota awareness scale, and that they could not fully establish the relationship between microbiota and diseases. In this context, the society should be sensitized on diseases that they are aware of, as well as daily nutritional habits that support the microbiota, so that participants can establish the relationship between microbiota and diseases and can help to eradicate these inadequacies. As a result, it is recommended that training programs targeting the relationship between microbiota and health in women planning pregnancy, such as "breast milk microbiota and infant nutrition," "microbiota and its importance in women's health," and "microbiota and disease relationship," be organized and encouraged to participate in.

## Data Availability

Data used and analyzed in the current study are available from the corresponding author upon reasonable request.
